# Prevalence and socio-demographic correlates of poor sleep quality among older adults in Hebei province, China

**DOI:** 10.1038/s41598-020-68997-x

**Published:** 2020-07-23

**Authors:** Yun-Shu Zhang, Yu Jin, Wen-Wang Rao, Yuan-Yuan Jiang, Li-Jun Cui, Jian-Feng Li, Lin Li, Gabor S. Ungvari, Chee H. Ng, Ke-Qing Li, Yu-Tao Xiang

**Affiliations:** 1 Hebei Provincial Mental Health Centre, Hebei Provincial Sixth People’s Hospital, Baoding, Hebei China; 2 Unit of Psychiatry, Centre for Precision Medicine Research and Training, Institute of Translational Medicine, Faculty of Health Sciences, University of Macau, 3/F, Building E12, Avenida da Universidade, Taipa, Macau SAR China; 3 Division of Psychiatry, School of Medicine, University of Western Australia, Perth, Australia; 4 University of Notre Dame, Australia, Fremantle, Australia; 5 Department of Psychiatry, The Melbourne Clinic and St Vincent’s Hospital, University of Melbourne, Richmond, VIC Australia; 6 Center for Cognition and Brain Sciences, University of Macau, Macao SAR, China

**Keywords:** Epidemiology, Population screening, Health care, Geriatrics, Public health, Quality of life

## Abstract

Poor sleep quality is associated with negative health outcomes and high treatment burden. This study investigated the prevalence of poor sleep quality and its socio-demographic correlates among older adults in Hebei province, which is a predominantly agricultural region of China. A large-scale cross-sectional epidemiological survey was conducted from April to August 2016. The study used a multistage, stratified, cluster random sampling method. Sleep quality was assessed by the Pittsburgh Sleep Quality Index (PSQI). A total of 3,911 participants were included. The prevalence of poor sleep quality (defined as PSQI > 7) was 21.0% (95% CI 19.7–22.2%), with 22.3% (95% CI 20.9–23.8%) in rural areas and 15.9% (95% CI 13.4–18.4%) in urban areas. Multivariable logistic regression analyses found that female gender (P < 0.001, OR 2.4, 95% CI 2.00–2.82), rural areas (P = 0.002, OR 1.5, 95% CI 1.14–1.86), presence of major medical conditions (P < 0.001, OR 2.4, 95% CI 2.02–2.96) and family history of psychiatric disorders (P < 0.001, OR 2.7, 95% CI 1.60–4.39) were independently associated with higher risk of poor sleep quality. Poor sleep quality was common among older adults in Hebei province of China. Regular assessment of sleep quality and accessible sleep treatments for older population should be provided in agricultural areas of China.

## Introduction

Older adults are more likely to suffer from poor sleep quality compared to younger adults due to more frequent physical and mental disorders^1^. More than half of older adults complain about poor sleep quality^2,3^, including increased awakenings, low sleep efficiency, poor subjective sleep quality and decreased night sleep duration^1,4^. Poor sleep quality is associated with negative health outcomes, such as fatigue, low quality of life, risk of medical and psychiatric comorbidities and even mortality^5–7^.

Sleep quality can be measured with both objective [e.g., polysomnography (PSG)], and subjective instruments [e.g., sleep diary and Pittsburgh Sleep Quality Index (PSQI)]^8^. Of these, the PSQI is the most widely used measure of global sleep quality which covers subjective sleep quality, sleep latency, sleep duration, sleep efficiency, sleep disturbance, daytime dysfunction, and use of sleep medications.

Determining the prevalence of poor sleep quality is important for health professionals and policymakers to understand its impact on population health and the need for appropriate preventive strategies and health resource allocations. In the past decade, epidemiological studies have examined the prevalence of poor sleep quality among older adults in various countries. For instance, the prevalence of poor sleep quality in older adults was 37.3% in Japan^9^, while the corresponding figure was 64.3% in Korea^10^. Several studies explored sleep quality among older adults in low- and middle-income countries in Africa, Asia and North America, with the prevalence ranging from 7.7% to 40.0%^11–13^. As the pattern of sleep problems including poor sleep quality is greatly influenced by sociocultural and economic factors^14,15^, the study of poor sleep quality prevalence across different countries is thus important. In China, the population aged 65 years and above increased to 8.2% in 2010 and the figure is expected to reach 23.3% by 2050^16^. Considering the negative impact of poor sleep quality on health, it is essential to examine the prevalence of poor sleep quality in older population in China. Previous studies have examined the patterns of poor sleep quality in Chinese older adults and the prevalence varied from 32.9% to 49.7%^13,17–22^. However, most studies on poor sleep quality in China were conducted in major cities, such as Beijing, Shanghai and Guangzhou^17,18,23,24^, while very limited data are available in agricultural areas.

Hence, we investigated the prevalence of poor sleep quality and its associated correlates in older adults in Hebei province, which is a predominantly agricultural area of China. We hypothesized that the prevalence of poor sleep quality in older adults in Hebei province would be different from previous findings obtained in major cities of China.

## Results

### Characteristics of participants

Of the 23,675 persons (≥ 18 years of age) invited to participate in the survey, 20,884 met the study entry criteria and completed the assessments, giving a participation rate of 88.2%. Finally, 3,911 participants (1,892 men and 2,019 women) aged ≥ 65 years were included in this study. Table 1 presents the socio-demographic characteristics of participants by sleep quality. Gender, marital status, residential area, education level, annual household income, presence of major medical conditions and family history of psychiatric disorders significantly differed between the good and poor sleep quality groups (all P values < 0.05). Table 2 shows PSQI total and component scores of the participants.

**Table 1:** Socio-demographic characteristics of the study population (N = 3,911) by sleep quality.

Variables	Whole sample (N = 3,911)		Those with good sleep quality (N = 3,091)		Those with poor sleep quality (N = 820)		Statistics		
	n	%	n	%	n	%	${\chi }^{2}$	df	P
**Age (years)**							1.0	1	0.32
65–74	2,754	70.4	2,165	70.0	589	71.8			
$\ge$ 75	1,157	29.6	926	30.0	231	28.2			
Male	1,892	48.4	1,639	53.0	253	30.9	127.6	1	**< 0.001**
Married/cohabiting	3,036	77.6	2,433	78.7	604	73.5	10.0	1	**0.002**
Urban area	826	21.1	695	22.5	131	16.0	16.5	1	**< 0.001**
**Education level**							25.7	1	**< 0.001**
Primary school or below^a^	2,640	67.5	2,026	65.5	614	74.9			
Secondary school or higher	1,271	32.5	1,065	34.5	206	25.1			
Unemployed	1,379	35.3	1,106	35.8	273	33.3	1.7	1	0.19
Low income^b^	3,016	77.1	2,340	75.7	676	82.4	16.5	1	**< 0.001**
Living alone	642	16.4	491	15.9	151	18.4	3.0	1	0.08
Religious beliefs	268	6.8	199	6.4	68	8.3	3.5	1	0.06
Health insurance	3,822	97.7	3,017	97.6	805	98.2	0.93	1	0.34
Major medical conditions^c^	2,572	65.8	1,911	61.8	661	80.6	101.6	1	**< 0.001**
Family history of psychiatric disorders	70	1.8	41	1.3	29	3.5	18.0	1	**< 0.001**

	Mean	SD	Mean	SD	Mean	SD	t	df	P
Age (years)	72.00	6.09	72.02	6.13	71.94	5.96	0.3	3,909	0.73

Bolded values: < 0.05; *M* mean, *PSQI* Pittsburgh Sleep Quality Index, *SD* standard deviation.

Good sleep quality was defined as Pittsburgh Sleep Quality Index (PSQI) < 7.

^a^Primary school or below = less than 7 years of education.

^b^Low income: annual household income < RMB30,000 (approximately USD4,242).

^c^Major medical conditions included hypertension, diabetes, cerebrovascular disease, cancer, and gastrointestinal diseases.

**Table 2:** PSQI total and component scores in all participants.

	Total (N = 3,911)		Male (N = 1,892)		Female (N = 2,019)		Rural (N = 3,085)		Urban (N = 826)	
	M	SD	M	SD	M	SD	M	SD	M	SD
PSQI total score	5.05	3.78	4.30	3.21	5.75	4.13	5.17	3.87	4.62	3.42
Subjective sleep quality	1.02	0.76	0.89	0.69	1.15	0.80	1.04	0.77	0.98	0.70
Sleep latency	1.05	1.06	0.83	0.96	1.25	1.10	1.07	1.07	0.97	1.00
Sleep duration	0.80	0.91	0.74	0.86	0.86	0.95	0.80	0.92	0.83	0.87
Sleep efficiency	0.33	0.80	0.24	0.67	0.42	0.89	0.36	0.82	0.20	0.68
Sleep disturbance	0.90	0.62	0.82	0.59	0.98	0.64	0.92	0.63	0.83	0.55
Daytime dysfunction	0.78	0.97	0.68	0.92	0.87	1.00	0.83	1.00	0.61	0.80
Use of sleep medication	0.16	0.61	0.10	0.47	0.22	0.70	0.17	0.61	0.16	0.58

*M* mean, *PSQI* Pittsburgh Sleep Quality Index, *SD* standard deviation.

### Prevalence of poor sleep quality

The prevalence of poor sleep quality (defined as PSQI > 7) was 21.0% (95% CI 19.7–22.2%). Table 3 shows prevalence of poor sleep quality by age, gender and region. The prevalence of poor sleep quality was 22.3% (95% CI 20.9–23.8%) in rural sample, with 14.1% (95% CI 12.3–15.8%) in men and 30.4% (95% CI 28.1–32.7%) in women. In contrast, the prevalence of poor sleep quality was 15.9% (95% CI 13.4–18.4%) in urban sample, with 10.5% (95% CI 7.4–13.7%) in men and 20.2% (95% CI 16.5–23.9%) in women. The prevalence of poor sleep quality in men was significantly lower than women in both rural and urban regions.

**Table 3:** Prevalence of poor sleep quality by age, gender and region.

Age (years)	Male % (95% CI)	Female % (95% CI)	Total % (95% CI)
**Whole sample**			
65–74	12.4 (10.6–14.1)	30.3 (27.9–32.7)	21.4 (19.9–22.9)
$\ge$ 75	16.0 (12.9–19.2)	23.2 (19.9–26.5)	20.0 (17.7–22.3)
Total	13.4 (11.8–14.9)	28.1 (26.1–30.0)	21.0 (19.7–22.2)
**Rural sample**			
65–74	12.9 (10.9–14.8)	32.9 (30.1–35.7)	22.7 (21.0–24.5)
$\ge$ 75	17.4 (13.6–21.1)	24.6 (20.7–28.5)	21.3 (18.6–24.0)
Total	14.1 (12.3–15.8)	30.4 (28.1–32.7)	22.3 (20.9–23.8)
**Urban sample**			
65–74	9.9 (6.1–13.7)	20.7 (16.0–25.3)	15.8 (12.7–18.9)
$\ge$ 75	11.8 (6.1–17.5)	19.3 (13.1–25.4)	16.0 (11.7–20.2)
Total	10.5 (7.4–13.7)	20.2 (16.5–23.9)	15.9 (13.4–18.4)

*CI* confidential interval.

### Correlates of poor sleep quality

Table 4 presents the independent correlates of poor sleep quality. Female gender (P < 0.001, OR 2.4, 95% CI 2.00–2.82), rural areas (P = 0.002, OR 1.5, 95% CI 1.14–1.86), presence of major medical conditions (P < 0.001, OR 2.4, 95% CI 2.02–2.96) and family history of psychiatric disorders (P < 0.001, OR 2.7, 95% CI 1.60–4.39) were independently associated with poor sleep quality.

**Table 4:** Independent correlates of poor sleep quality by multiple logistic regression analysis.

Variables	Multivariate regression analysis		
	OR	95% CI	P value
Female gender	2.4	2.00–2.82	**< 0.001**
Rural region	1.5	1.14–1.86	**0.002**
Married/cohabiting	0.9	0.78–1.14	0.539
Primary school or below^a^	1.2	0.97–1.43	0.100
Low income^b^	1.1	0.87–1.39	0.429
Major medical conditions^c^	2.4	2.02–2.96	**< 0.001**
Family history of psychiatric disorders	2.7	1.60–4.39	**< 0.001**

Bolded values: < 0.05; *CI* confidential interval, *OR* odds ratio.

^a^Primary school or below = less than 7 years of formal education.

^b^Low income: annual household income < RMB30,000 (approximately USD4,242).

^c^Major medical conditions included hypertension, diabetes, cerebrovascular disease, cancer, and gastrointestinal diseases.

## Discussion

This large-scale epidemiological study found that the prevalence of poor sleep quality (defined as PSQI > 7) was 21.0% (95% CI 19.7–22.2%) in Chinese older adults, which is significantly associated with female gender, rural areas, presence of major medical conditions and family history of psychiatric disorders.

A previous study found that 15.7% of 7,154 older adults aged ≥ 60 years in China reported moderate and severe sleep problems as measured by a question about sleep quality^13^. Another study of older adults (aged 50–70 years) in Beijing and Shanghai found that 16% of 3,289 participants reported poor sleep quality (19% Beijing vs. 13% Shanghai) as measured by self-reported sleep duration^18^. In a study of older adults aged ≥ 65 years in 22 provinces in China, 35% of 15,638 participants reported “fair to very bad” sleep quality according to a question on “how do you rate your sleep quality recently?”^22^. However, due to the use of different measures on sleep quality, direct comparisons between results could not be conducted.

The prevalence of poor sleep quality among the elderly in this study (21.0%, 95% CI 19.7–22.2%) is lower compared to most of other studies using PSQI in both China and other countries, such as Japan (37.3%)^9^, and Korea (64.3%)^10^. The prevalence of poor sleep quality (PSQI > 5) among the older adults (aged ≥ 60) was 47.1% in Hong Kong^25^, while the corresponding figure (aged ≥ 65) was 49% in Taiwan^20^. In mainland China, the prevalence of poor sleep quality among older adults (aged ≥ 60) was 41.5% (95% CI 38.6–44.5%) in Shanghai^23^, and 49.7% in Anhui province^21^. In addition, the PSQI total score in this study was 5.05 $\pm$ 3.78, which is also lower than the corresponding figures in Taiwan (6.3 $\pm$ 4.4)^20^, Anhui province (7.74 $\pm$ 3.06) and South Korea (6.42 $\pm$ 3.60)^10^. The lower prevalence of poor sleep quality in this study compared to other studies could be possibly explained by several reasons. First, different definitions of ‘older adults’ (e.g., ≥ 60 years^21,23,25^ and ≥ 65 years^9,10,20^) and definitions of ‘poor sleep quality’ (e.g., PSQI total scores of ≥ 5^10,20,23,25^, ≥ 6^9^, > 7^21^) were applied in different studies. Second, different study periods, sample sizes, sampling methods (one stage vs. multi-stage), interview techniques (face-to-face vs. telephone interview) and statistical methods (e.g., univariate vs. multivariate analyses) may result in different findings. Finally, Hebei province is a predominantly agricultural area, with less urbanization and industrialization compared to major cities involved in previous studies, therefore, residents in Hebei may have less daily living pressures, which could in turn reduce the likelihood of poor sleep quality.

Poor sleep quality was more common in older women than in men, which supports previous findings^26–28^. Generally, older women often have heavy household responsibilities and burden in China, which can lead to higher risk of sleep problems and depression^29,30^. In addition, women may be more sensitive to negative life events, such as the loss of family members or friends, which may result in poor sleep quality^24^. Furthermore, in China older women usually have lower income, fewer social activities, and poorer social support compared to men, all of which are associated with higher risk of poor sleep quality^31^.

Higher prevalence of poor sleep quality in rural (22.3%) than urban (15.9%) residents is consistent with earlier findings^27^. Older adults in rural areas are burdened with heavy farm work and physical pain or other health problems, which may affect sleep quality^32^. Furthermore, inadequate access to healthcare services and limited awareness of sleep hygiene in rural regions may increase the risk of sleep problems^33^. Similar to earlier findings, the presence of major medical conditions was significantly associated with poor sleep quality. In this study, major medical conditions found included hypertension, diabetes, cerebrovascular disease, cancer, and gastrointestinal diseases, all of which could lead to depression, anxiety and poor sleep quality^1,34,35^. Moreover, adverse effects of certain medications for major medical conditions could also lead to poor sleep quality^1^. Subjects with family history of psychiatric disorders were more likely to suffer from psychiatric disorders, which, in turn, could worsen sleep quality. Moreover, sleep problems were significantly associated with psychiatric disorders. For example, studies found that sleep problems could be a prodromal symptom of some psychiatric disorders, such as depression^36–38^, and there was a high rate of comorbidity between sleep problems and some psychiatric illness, particularly mood and anxiety disorders^39^. All these factors could explain the association between poor sleep quality and family history of psychiatric disorders.

The strengths of this study included the inclusion of agricultural region, large sample size, multistage random sampling, and use of standardized instrument on sleep quality. However, there are several limitations to this study. First, this was a cross-sectional study, thus the causal relationship between poor sleep disturbances and other variables could not be verified. Second, people who could not understand the content of assessments were not included, which could lead to potential selection bias. Third, use of medications for sleep problems, such as sleeping pills, were not recorded. Fourth, poor sleep quality was only measured by a self-administered instrument, rather than objective measures.

In conclusion, poor sleep quality was common among older adults in Hebei province of China, although the prevalence was relatively lower than that in most studies in China and other countries. In order to reduce the negative impact of poor sleep quality on health outcomes, regular assessment of sleep quality and accessible sleep treatments for older population should be provided in agricultural areas of China.

## Methods

### Subjects and sampling

This study, conducted from April 1 to August 31, 2016, was part of a large-scale cross-sectional epidemiological survey on mental health in Hebei province, China^40^. The study protocol was approved by the Human Research Ethics Committee of Hebei Mental Health Centre. All methods were performed in accordance with the relevant guidelines and regulations.

The sample size was calculated using the program OpenEpi by the formula: $n=[DEFF * Np(1-p)]/[(d^2/(Z_{(1-\alpha/2)^2}) * (N-1) + p*(1-p))]$^41^. Given the finding of a previous survey in Hebei province (p; the prevalence of any type of psychiatric disorders: 18.51%)^42^, design effect of 2.0, significance level of 99% (two-tailed), and the precision of the estimate of 0.1p (d), the sample size should be at least 20,013. With assumed response rate of 80%, a total of 24,000 participants should be included.

The inclusion criteria were: (1) aged 65 or above; (2) permanent residents in Hebei province; (3) ability to understand the content of the assessment; and (4) willingness to participate in this study. Written informed consent was obtained from all participants. A multistage, stratified, cluster random sampling method was used in this study^40^. In Hebei province, neighborhood communities and villages each with several hundreds of households are the basic community units in urban and rural areas, respectively. The sampling process included first, all the eleven administrative regions of Hebei province were included in this survey. Urban and rural residents were categorized according to the household registration system in local public security departments. Second, following an earlier epidemiological survey in Hebei province^42^ and taking into consideration the population ratio of urban to rural areas, 1–4 districts and 1–7 towns were randomly selected by a computer-generated random number table in each administrative region. Finally, this survey included 20 communities in urban regions and 58 villages in rural regions, from which 23,675 eligible residents were randomly selected and invited to join this study during the study period.

### Assessment tools and procedure

Basic socio-demographic and clinical characteristics, such as age, gender, marital status, residential area, education level, employment status, annual household income, co-living status, religious beliefs, health insurance, presence of major medical conditions and family history of psychiatric disorders, were collected using a data collection sheet designed for this study. Following previous studies^31,36^, marital status was dichotomized: married/cohabiting and others (e.g., single, divorced and widowhood).

Sleep quality was assessed by the validated Chinese version of the PSQI^8,43^. The PSQI is a self-administered scale to measure sleep quality over the past 1-month, with 19 items covering seven domains, including subjective sleep quality, sleep latency, sleep duration, sleep efficiency, sleep disturbance, daytime dysfunction, and use of sleep medications. Each item is scored from 0 to 3, with a higher score indicating lower sleep quality. A PSQI total score of ≥ 7 indicates “poor sleep quality” with a sensitivity of 98.3% and specificity of 90.2%^43^.

### Statistical analysis

Epi data software (Version 3.1, Odense, Denmark) was used to establish the database. Statistical analyses were conducted using SPSS, Version 24.0 (IBM SPSS, IBM Crop., Armonk, NY, USA). Comparisons between good and poor sleep quality groups were conducted using Chi-square test and two independent samples t-test, as appropriate. The normal distribution of continuous variables was checked using Kolmogorow–Smirnov test. Multiple logistic regression analysis with the “enter” method was used to examine the independent correlates of poor sleep quality. Poor sleep quality was entered as the dependent variable, while those that significantly differed between the two groups in the univariate analyses were entered as independent variables. Significant level was set at 0.05, with two-sided tests.

## Data Availability

The Clinical Research Ethics Committee of Hebei Mental Health Hospital that approved the study prohibits the authors from making the research data set publicly available. Readers and all interested researchers may contact Dr. Keqing Li (Email address: likel002@sina.com) for details. Dr. Li could apply to the Clinical Research Ethics Committee of Hebei Mental Health Hospital for the release of the data.
